# The Importance of Role Models in Research

**DOI:** 10.1371/journal.ppat.1005426

**Published:** 2016-06-02

**Authors:** James B. Bliska

**Affiliations:** Department of Molecular Genetics and Microbiology, Center for Infectious Diseases, Stony Brook University, Stony Brook, New York, United States of America; University of Florida, UNITED STATES

I have been extremely fortunate to have a number of exceptional role models during my research career. By emulating these individuals, I gained the skills and the knowledge that I needed to achieve a successful research career in the field of bacterial pathogenesis. It is not surprising that my PhD dissertation advisor at Berkeley, Nicholas Cozzarelli, and my postdoctoral advisor at Stanford, Stanley Falkow, both highly eminent and successful scientists, were important role models. However, as I look back, it becomes clear that my interactions with mentors when I was an undergraduate were of equal importance in my scientific development. Below I describe some of my experiences as an undergraduate, and I hope that this story will serve as a reminder of the importance of role models for young scientists.

My research career got its start at a bus stop on a bitterly cold morning in the winter of 1980 in Madison, Wisconsin. I was on my way to the University of Wisconsin, where I was an undergraduate, to interview for a research aid position at a laboratory. A middle-aged woman at the bus stop commented to me that I should be wearing a hat and gloves on such a cold morning. I politely thanked her for her advice, but was secretly irritated, in part due to my nervousness about the upcoming interview. When I arrived at the laboratory to meet with a secretary for the interview, who was sitting behind the desk in the office? The woman from the bus stop! Her name is Frances Mann and, after I was hired, Frances often joked that she gave me the job because I needed the money to buy a hat and gloves. She also enjoys taking full credit for starting my research career.

By sheer luck I was hired into the laboratory of Oliver Smithies, who would go on to win a Nobel prize in physiology or medicine in 2007 for his role in discovering how to modify genes in mice using embryonic stem cells. I was initially employed to wash glassware, but soon was given other tasks, including purifying recombinant plasmids from *Escherichia coli*. These were the early days of recombinant DNA and “plasmid preps” were done in high-containment suites and took days to complete. Supercoiled plasmid DNA was isolated using equilibrium density centrifugation in a cesium chloride–ethidium bromide solution. In one of the last steps, in a darkened room, a UV light was used to illuminate the band of supercoiled plasmid DNA in the centrifuge tube, which was collected with a pipette. Siphoning off that glowing band of supercoiled plasmid DNA was exciting and satisfying, and the experience likely contributed to my long-standing interest in the biology of bacteria and their plasmids. It was in this environment that I encountered my first scientific role model, a technician in the laboratory named Natalie Borenstein. By following Natalie’s example, I learned to always follow good sterile technique, to never cut corners, and to pay close attention to details. Students who have passed through my laboratory can attest to my attention to detail, and they can thank Natalie for this attribute.

Eventually I became immersed in the laboratory, attended group meetings, and pursued a research project for my undergraduate honors thesis. Nobuyo Maeda, an outstanding postdoc in the laboratory, guided my research and became a second influential role model. With Nobuyo’s help I was able to determine the sequence of 3.1 kb of a Chimpanzee globin gene by the Maxam and Gilbert technique. From her example, I learned how to analyze and publish data, and she taught me that success in science requires dedication and hard work.

The highlight of my research experience as an undergraduate was when I was placed at a bench space adjacent to Oliver’s. Although he had a conveniently located office, it was always empty because Oliver preferred to spend all of his time at the bench. Whether he was running experiments, writing manuscripts, or discussing results, he was always in the laboratory. Oliver had an enormous impact on me and, as a result, I decided to apply to graduate school. From his example, I learned that a successful scientist can have boundless enthusiasm for research and a balanced approach to life. Oliver is fond of saying, “Make your first love your hobby and your second love your career.” His first love is flying. When Oliver invited me to fly in a glider with him one day, I jumped at the chance. That was an experience I will never forget.

In my own laboratory I have made an effort to hire undergraduate research aides. I try to foster a laboratory environment in which all members (technicians, graduate students, postdocs, and principal investigators) can serve as scientific role models. It seems to be working, as several of these research aides have developed into outstanding undergraduate researchers and have gone on to obtain PhDs or MD/PhDs and successful careers in science.

**Image 1:**
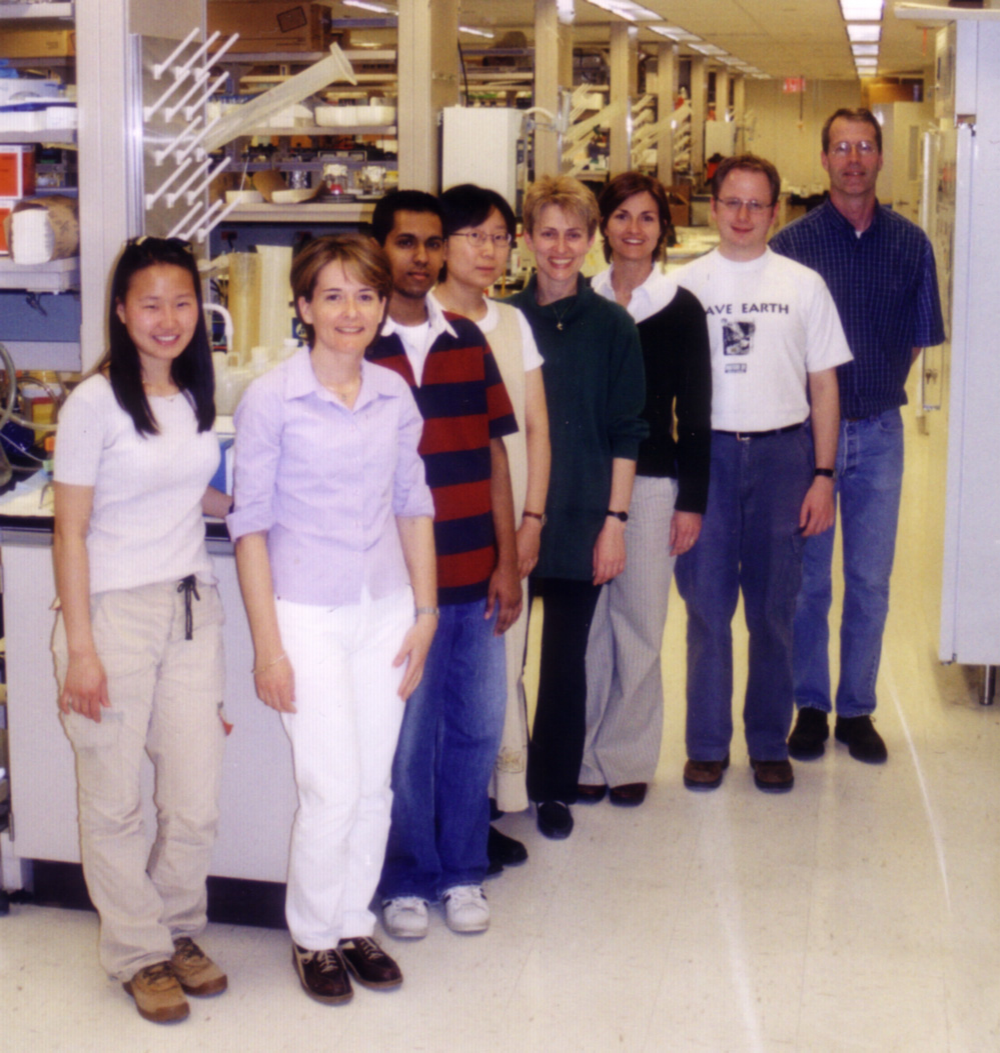
Bliska laboratory members circa 2005. Left to right: Hachung Chung, Celine Pujol, Ryan Rampersaud, Yue Zhang, Maya Ivanov, Gloria Viboud, Jens Grabenstein, James Bliska. Hachung and Ryan were laboratory aides that that became outstanding undergraduate researchers and then went on to obtain a PhD at Harvard and a MD/PhD at Columbia, respectively. Hachung is currently a postdoc at Rockefeller University and Ryan is a resident in psychiatry at UCSF. Photo provided by Michelle Ryndak.

